# Comparative Genomic Analysis of the Genus *Nocardiopsis* Provides New Insights into Its Genetic Mechanisms of Environmental Adaptability

**DOI:** 10.1371/journal.pone.0061528

**Published:** 2013-04-23

**Authors:** Hong-Wei Li, Xiao-Yang Zhi, Ji-Cheng Yao, Yu Zhou, Shu-Kun Tang, Hans-Peter Klenk, Jiao Zhao, Wen-Jun Li

**Affiliations:** 1 Key Laboratory of Microbial Diversity in Southwest China, Ministry of Education and the Laboratory for Conservation and Utilization of Bio-resources, Yunnan Institute of Microbiology, Yunnan University, Kunming, People’s Republic of China; 2 College of Biological Resources and Environment Science, Qujing Normal University, Qujing, People’s Republic of China; 3 State Key Laboratory of Microbial Resources, Institute of Microbiology, Chinese Academy of Sciences, Beijing, People’s Republic of China; 4 Zhejiang Province Key Laboratory for Food Safety; Institute of Quality and Standard for Agro-products, Zhejiang Academy of Agricultural Sciences, Hangzhou, People’s Republic of China; 5 Deutsche Sammlung von Mikroorganismen und Zellkulturen, Braunschweig, Germany; 6 Shenzhen Key Laboratory of Bioenergy, Beijing Genomics Institute at Shenzhen (BGI-Shenzhen), Shenzhen, People’s Republic of China; 7 Key Laboratory of Biogeography and Bioresource in Arid Lands, Xinjiang Institute of Ecology and Geography, Chinese Academy of Sciences, Ürűmqi, People’s Republic of China; Université Claude Bernard - Lyon 1, France

## Abstract

The genus *Nocardiopsis*, a widespread group in phylum Actinobacteria, has received much attention owing to its ecological versatility, pathogenicity, and ability to produce a rich array of bioactive metabolites. Its high environmental adaptability might be attributable to its genome dynamics, which can be estimated through comparative genomic analysis targeting microorganisms with close phylogenetic relationships but different phenotypes. To shed light on speciation, gene content evolution, and environmental adaptation in these unique actinobacteria, we sequenced draft genomes for 16 representative species of the genus and compared them with that of the type species *N. dassonvillei* subsp. *dassonvillei* DSM 43111^T^. The core genome of 1,993 orthologous and paralogous gene clusters was identified, and the pan-genomic reservoir was found not only to accommodate more than 22,000 genes, but also to be open. The top ten paralogous genes in terms of copy number could be referred to three functional categories: transcription regulators, transporters, and synthases related to bioactive metabolites. Based on phylogenomic reconstruction, we inferred past evolutionary events, such as gene gains and losses, and identified a list of clade-specific genes implicated in environmental adaptation. These results provided insights into the genetic causes of environmental adaptability in this cosmopolitan actinobacterial group and the contributions made by its inherent features, including genome dynamics and the constituents of core and accessory proteins.

## Introduction

The genus *Nocardiopsis* is affiliated with the phylum Actinobacteria, which is marked by being Gram-positive and having a genome with a high guanine and cytosine (G+C) content. This unique group has previously received attention because of the pathogenicity of the type species, *N. dassonvillei* subsp. *dassonvillei* [1]–[3]. Moreover, the genus is of interest for both its ecological versatility and its ability to produce a rich array of bioactive metabolites. Numerous studies have shown that *Nocardiopsis* strains are ubiquitously distributed across a diverse range of environments, such as saline or alkaline habitats, deserts, marine habitats, plant tissues, animal guts, and indoor environments [4], [5]. Members of the genus also produce such bioactive metabolites as methylpendolmycin [6], apoptolidin [7], griseusin D [8], lipopeptide biosurfactants [9], thiopeptides [10] and naphthospironone A [11]. Undoubtedly, the outstanding and diverse physiological traits of microbial populations can be attributed to their underlying genetic diversity and also their mechanisms of generating genetic variation.

A key concept emerging from the current genomics era is the partitioning of the microbial genome into “core” and “accessory” elements [12], together called the pan-genome [13]. The former includes those genes responsible for the essential housekeeping functions of the cell and defines the “essence” of a given taxonomic unit by excluding genes not present in all strains. Accessory elements, in contrast, include those genes not found in all strains, either because they were acquired through horizontal gene transfer or because they were differentially lost. Although the functions of these genes tend to be less clear, generally they are thought to extend the physiological and ecological capabilities of the microbial cells [14]–[17]. Usually, microbial genomes evolve dynamically by both losing and gaining genes. Genome reduction is considered an evolutionary feature of intracellular pathogenic bacteria, in which gene loss is more likely to occur than gene gain [18]–[20]. Differential gene losses help create new species, and the evolutionary loss process has been investigated in many studies [19]–[23]. Gene gain is also an important evolutionary force, especially in ecologically-versatile species. However, how highly-adaptable species, such as members of *Nocardiopsis*, maintain the genomic flexibility to survive across such a broad range of ecologies is not yet known.

With recent advances in next-generation sequencing technology, massive amounts of genetic data are helping to revolutionize our understanding of the ecology and diversity of microorganisms in natural settings. Comparative genomics and phylogenomics provide new approaches to elucidate their adaptations to diverse environments and their genetic evolution [23]–[25]. Currently, complete genomes have been sequenced for the type species (*N. dassonvillei* subsp. *dassonvillei* DSM 43111) and one strain (*N. alba* ATCC BAA-2165) [26], [27] in the genus *Nocardiopsis*, which comprises 35 validly-described species [5]. In this study, we determined the genome sequences of another 16 representative species and performed comparative genomic analyses with *N. dassonvillei* subsp. *dassonvillei* DSM 43111^T^ to investigate the evolutionary history and genetic basis of environmental adaptability.

## Results and Discussion

### Genomic Features

Together with *N. dassonvillei* subsp. *dassonvillei* DSM 43111^T^, the 17 type strains studied were dispersed widely across the phylogenetic tree based on the 16S rRNA gene sequences and therefore are considered to well represent the species diversity in the genus (Figure S1). The genomic G+C content of test species averaged around 70%. The lowest genomic G+C content was found in *N. alkaliphila*, with 67.5%, and the highest in *N. potens*, with 74.8% (Table S1). Genomic G+C content is a result of mutation and selection [28] involving multiple factors, including environment, symbiotic lifestyle, aerobiosis, nitrogen fixation ability, and the combination of polIII α subunits [29]. In addition, the genome sizes of these species ranged from 4.9–7.4 Mbp, and the number of protein-coding genes ranged from 4,848–6,907 (Table S1). A larger genome size usually correlates with a more complex habitat and enables microorganisms to cope with such conditions more easily, as it encodes a larger metabolic and stress-tolerance potential [30]. However, various hypotheses also argue that genome size is itself subject to natural selection, i.e., the tight packing and small sizes of bacterial genomes is an adaptation for reproductive efficiency or competitiveness [31]. Clearly, a balance exists between maintaining a minimal genome size and the need to respond to, or exploit, environmental conditions.

### Core and Pan-genome Analysis

**Figure 1:**
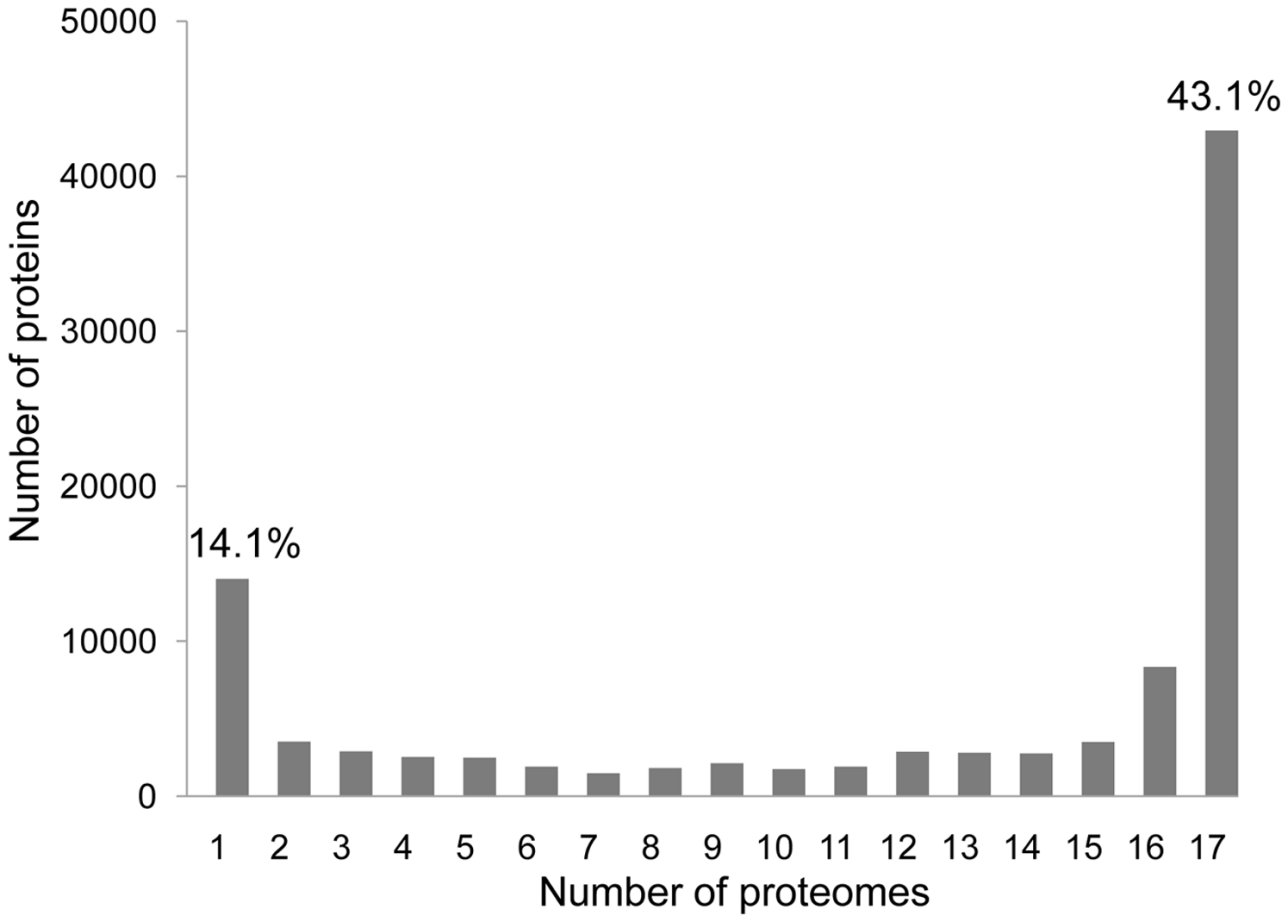
Occurrence of individual proteins in 17 *Nocardiopsis* proteomes ranged from one (a species-specific protein) to 17 (a core protein). At the far left of the *x*-axis are species-specific proteins found in a single proteome (14,019 proteins; 14.1% of the total proteomes), while at the opposite end of the scale are *Nocardiopsis* core proteins found in all 17 proteomes (42,943; 43.1%).

Using the reciprocal best hit method, 99,684 protein coding sequences belonging to 17 predicted proteomes of *Nocardiopsis* were grouped into 22,143 homologous clusters, including 14,019 clusters unique to one proteome (Table S2). Of the 99,684 proteins, the majority had homologous counterparts; however, some proteins were unique and could not be matched to any homologs in the pan-genome of *Nocardiopsis*. The highest percentage of unique proteins (25.1%) was observed in the proteome of *N. gilva* and the lowest (3.4%) in that of *N. dassonvillei* subsp. *dassonvillei* (Table S1). On average, 13.9% of the *Nocardiopsis* proteomes comprised unique proteins. We examined the distribution of the 22,143 homologous clusters across the 17 predicted proteomes and found that their distribution was bimodal, with most of the clusters present either in 16–17 proteomes or in only one (Figure 1). The 14,019 proteins distributed in only one *Nocardiopsis* proteome were inferred to be unique proteins. The 42,943 proteins could be assigned to 1,993 core clusters. The percentage of the genome that could be assigned to such core clusters ranged from 38.5% in *N. synnemataformans* to 49.1% in *N. xinjiangensis*; these genes represent the portion of the genome that is expected to be important or essential in all species of *Nocardiopsis* (Tables S1, S2).

**Figure 2:**
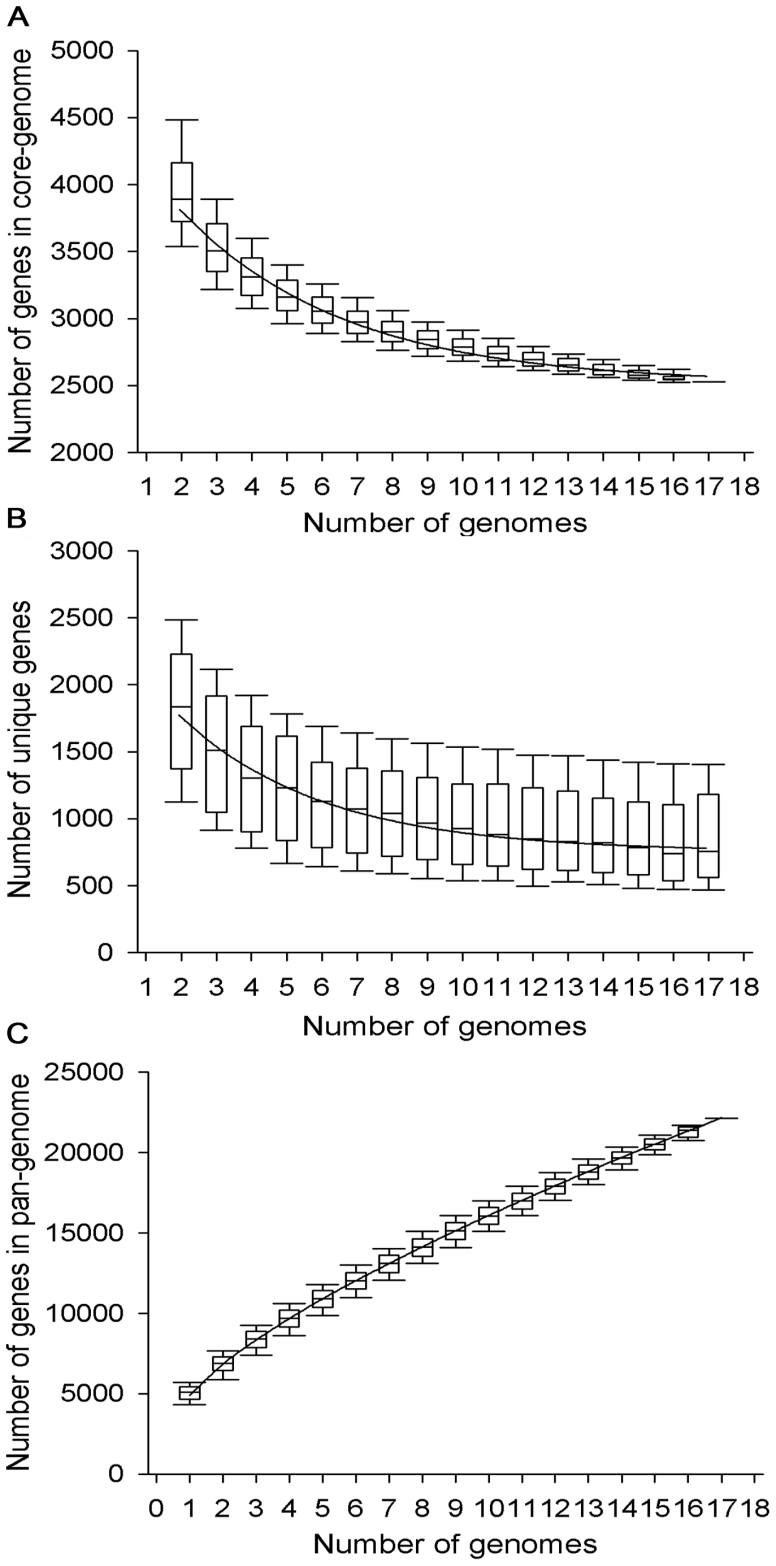
*Nocardiopsis* core, unique, and pan-genome evolution according to the number of sequenced genomes. The upper and lower edges of the boxes indicate the first (25th percentile of the data) and third (75th percentile) quartiles, respectively. The central horizontal line indicates the sample median (50th percentile) of 1,000 different random input orders of the genomes. The central vertical lines extend from each box as far as the data extend, to a distance of at most 1.5 interquartile ranges (i.e., the distance between the first and third quartile values). (A) Number of genes in common for a given number of genomes of different species. The exponential decay model based on the median value for the conserved core genes shows that the core-genome had a minimum of 2,517 genes (11% of the pan-genome). (B) Number of unique genes for a given number of genomes of different species. Decreasing numbers of unique genes per genome with increasing numbers of genomes was examined. The curve shows the exponential decay model based on the median value for unique genes when increasing numbers of genomes were compared. About 755 new unique genes will be added to the pan-genome for every new species genome sequenced, according this model. (C) Total number of non-orthologous genes for a given number of genomes of different species. With 17 sequenced genomes, the pan-genome has 22,143 total genes. The *Nocardiopsis* pan-genome is open and its size grows with the number of independent species sequenced.

To estimate the number of genes in each *Nocardiopsis* core genome, the number of shared genes found in the sequential addition of each new genome sequence was analyzed during 1,000 different random input orders of the genomes. As expected, the number of shared genes initially decreased with the addition of each new genome (Figure 2A). The *Nocardiopsis* genomes contained 5,864±640 genes (mean ± standard deviation), and the core genome contained 2,526±109 genes (Table S1). Nevertheless, the extrapolated curve indicated that the core genome reached a minimum of 2,517±32 genes, which would remain relatively constant even if additional type strain genomes were included. Previous comparison of 17 *Escherichia coli* genomes identified approximately 2,200 core genes [32]. Chen and his colleagues also used bioinformatic methods to determine that the core genome size of eight *E. coli* genomes was 2,865 genes [33], while the number of core genes in eight genomes of group B *Streptococcus* was 1,806 [34]. Theoretically, the core genome size is correlated with population size; genus-level populations should possess a smaller core genome than species-level populations. In practice, however, the core genome is also affected by the phylogenetic relationships among individuals, the genome size, and the inclusion threshold of conserved genes [32].

To determine the pan-genome of *Nocardiopsis* (global gene repertoire), the number of new unique genes added by each genome was also estimated during 1,000 different random input orders of genome. With each addition, the size of the pan-genome increased, even when the final genome was added. The average number of new genes supplied by a novel genome was 1,809 for the second genome added, and 840 for the last. As with the shared genes, a plot of the number of new genes was fitted well by a decaying exponential. We therefore applied the exponential decay model to identifying unique genes using the median value. Remarkably, this model estimated that 755±25 new genes were added per new genome (Figure 2B). Thus, the *Nocardiopsis* pan-genome is thought to be open.

Our calculations suggested that the genus *Nocardiopsis* has a gene reservoir of more than 22,000 genes (Figure 2C). Previous work estimated that each additional genome added an average of 33 new genes to the pool in group B *Streptococcus* and 27 new genes for group A *Streptococcus*, implying an open pan-genome. In the case of *Bacillus anthracis*, however, the number of species-specific genes added to the pan-genome dropped to zero after adding a fourth strain [34]. Additionally, Rasko and colleagues used the same methods to determine that about 300 new genes would be added with each new *E. coli* genome sequenced [32]. An open pan-genome implies that the group is still evolving by gene acquisition and diversification. The value of about 755 new genes in *Nocardiopsis* is exceptionally large, indicating incredible diversity and variability in these species and great adaptive potential.

In our data set, the genome of *Nocardiopsis* species contained, on average, 5,864 genes; the core genome contained 2,517 genes, and the pan-genome contained 22,143. In other words, random sampling of one gene within a randomly-selected *Nocardiopsis* genome had only a 43% probability of revealing a ubiquitous gene. On the other hand, whole-genome sequencing of one *Nocardiopsis* strain allows observation of only a quarter of the observed pan-genome, implying that further sampling of *Nocardiopsis* genomes is unlikely to change our estimate of the core genome significantly. However, the pan-genome is far from fully elucidated, and no single species can be regarded as fully representative of the genus.

### Functional Classification of Homologous Clusters

To understand the functions of the homologous clusters, we analyzed them according to the clusters of orthologous groups (COGs) functional categories (Table S3). However, for one fifth of the core clusters, function could not be determined. Of the 1,993 core clusters present in all 17 *Nocardiopsis* species, 393 appeared to be unique to the genus based on their absences in the COG databases. These unique core groups are candidate signature proteins for *Nocardiopsis*, which must include genes responsible for the main features of *Nocardiopsis* (e.g., its typical branched mycelium that fragments into rod-shaped and coccoid elements, and abundant aerial hyphae that frequently form a zigzag morphology).

The functional categories of the core paralogous clusters were analyzed in more detail, and the functions of the top ten paralogous clusters are shown in Table 1. In total, these clusters contained 7,745 genes, which account for around half of all the core paralogous genes. The TetR family of transcriptional regulators was the largest protein family in *Nocardiopsis* species, containing 47 genes on average. The TetR family controls genes whose products are involved in multi-drug resistance, antibiotic biosynthesis, efflux pumps, and osmotic stress. Members of the TetR family are particularly abundant in microbes, such as soil microorganisms and methanogenic bacteria, that are exposed to environmental changes and do not appear in intracellular pathogens and endosymbionts [35]. The second largest family was XRE transcription regulators. Members of the XRE family are involved in a wide range of gene regulation activities, including plasmid copying, restriction and modification systems, bacteriophage transcription control, and stress responses [36]. One typical class of XRE is the MmyB family, which comprises transcriptional factors with many homologs, is found predominantly in actinobacteria, and whose members are thought to play important roles in secondary-metabolite and fatty-acid metabolism [37]. The LuxR family is primarily involved in quorum sensing, biosynthesis, and metabolism, and the MerR family, which functions in detoxification and resistance, is mainly triggered by heavy metals, antibiotics, and oxidative stress [36], [38].

**Table 1:** Distribution of top ten functional categories among core paralogous proteins in 17 *Nocardiopsis* genomes.

Genome	Functional categories													
	Transcriptional regulator					ABC transporter	shortchain dehydrogenase	MajorFacilitatorsuperfamily	serine/threonineproteinkinase	hydrolase	methyltransferase	signaltransductionhistidinekinase	NRPSs/PKSs	cytochromeP450
	TetR	XRE	LuxR	MerR	other families*^a^*									
*N. alba*	43	12	16	14	72	101	28	33	29	23	24	18	15	9
*N. alkaliphila*	27	14	14	5	61	98	25	21	21	19	18	11	10	11
*N. baichengensis*	46	23	17	9	85	107	30	24	16	25	22	18	16	9
*N. chromatogenes*	50	23	17	12	92	125	31	33	19	30	21	18	24	10
*N. dassonvillei*	60	19	18	14	77	123	48	27	26	26	23	18	14	12
*N. ganjiahuensis*	62	18	20	17	96	161	45	36	34	28	27	22	10	17
*N. gilva*	45	17	12	13	70	94	21	26	13	17	19	13	11	10
*N. halophila*	49	19	17	12	90	106	32	28	21	26	20	17	11	10
*N. halotolerans*	49	15	18	12	80	112	40	30	27	25	27	18	14	13
*N. kunsanensis*	27	13	14	5	51	90	25	18	19	18	18	15	10	10
*N. lucentensis*	47	21	15	8	76	101	34	23	29	19	21	13	17	7
*N. potens*	67	20	21	16	83	108	33	36	29	31	27	24	18	9
*N. prasina*	44	16	12	14	87	129	43	28	21	21	21	14	11	12
*N. salina*	32	11	9	3	64	107	31	16	23	21	20	10	9	10
*N. synnemataformans*	60	27	23	20	86	132	45	30	29	23	22	18	17	13
*N. valliformis*	52	25	18	13	83	145	35	34	46	24	22	12	22	16
*N. xinjiangensis*	31	14	14	7	57	105	25	17	18	21	20	16	15	7

* The other families include ArsR, LacI, MarR, AraC, SARP, IclR, PadR, PucR, CopY, winged helix family, Crp/Fnr, and RpiR transcription factor.

One of the largest gene families found in *Nocardiopsis* coded for ABC transporters, and the total number of genes coding for these core proteins was 1,944, the number per species ranged from 90 to 161, with an average of 114 genes (Table 1). In further analyses, these genes were found to be associated with carbohydrate, amino acid, peptide, and iron-chelate uptake, as well as drug export (Table S4). ABC systems are involved not only in the import and export of a wide variety of substances, but also in many cellular processes. Studies have shown that intracellular parasites have fewer ABC transporters; in contrast, soil bacteria such as *Agrobacterium tumefaciens* and *Mesorhizobium loti* have more than 200 [39]. *Escherichia coli* and *Bacillus subtilis* have only about 80 and 84 ABC transporters, respectively [39], [40]. The major facilitator superfamily (MFS) represents the largest group of secondary active membrane transporters, which drive substrate translocation by exploiting the free energy stored in the chemiosmotic ion or solute gradients generated [41]. There are so many primary and secondary transporters in *Nocardiopsis* that their cells are able to modulate expression of each type of transporter for a particular substrate according to cellular and environmental needs.

Many gene clusters encoding the biosynthesis of polyketides and non-ribosomal peptides, members of the largest families of natural products, were found in the *Nocardiopsis* genomes, providing the potential to produce natural products and analogs. The cytochrome P450 superfamily catalyzes the oxidation of organic substances, including metabolic intermediates such as lipids and steroidal hormones, as well as xenobiotic substances such as drugs and other toxic chemicals. The cytochrome P450 superfamily includes major enzymes involved in drug metabolism and bioactivation, accounting for about 75% of the total number of different metabolic reactions [42]. Bioactivated metabolites in *Nocardiopsis* have important ecological functions linked to competition, intraspecies communication, and the viability and adaptability of *Nocardiopsis* in its external environment.

### Phylogenomic Analysis

**Figure 3:**
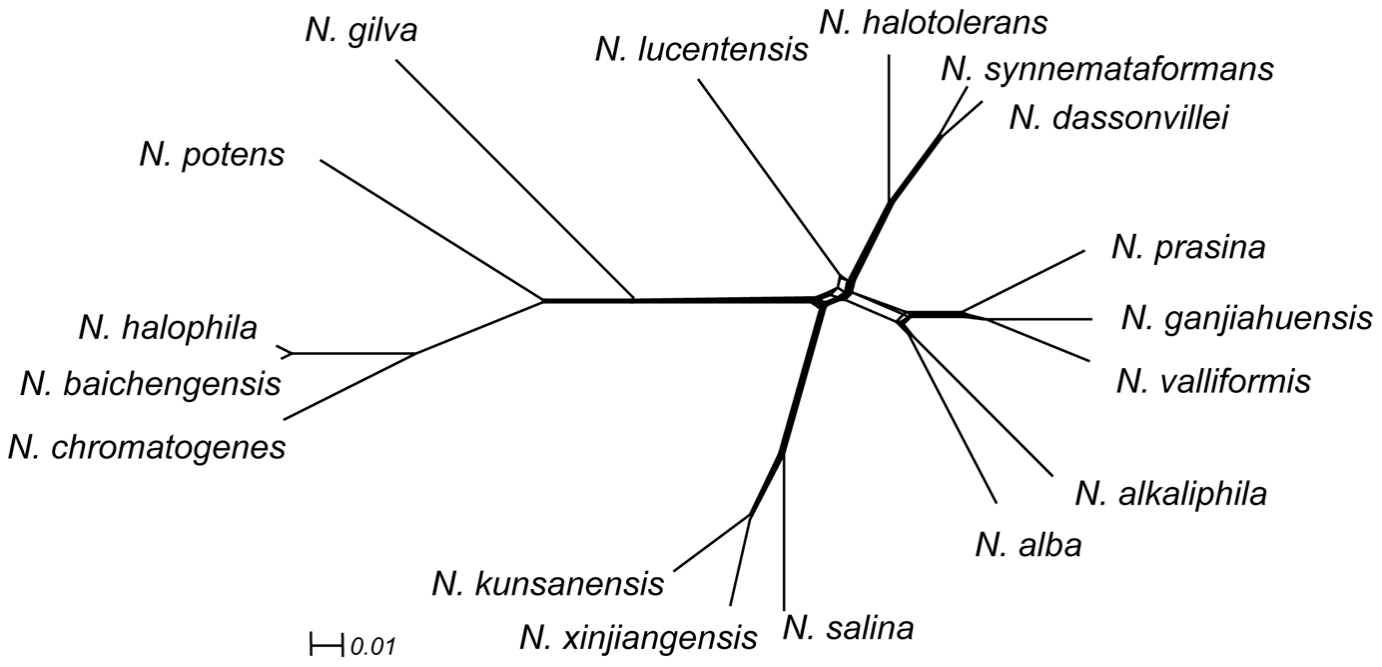
Phylogenetic network of 17 species of *Nocardiopsis*. The phylogenetic network was constructed with SplitsTree software [66], using a concatenated alignment of 1,555 orthologous core proteins as the input. The horizontal bar indicates number of substitutions per site.

**Figure 4:**
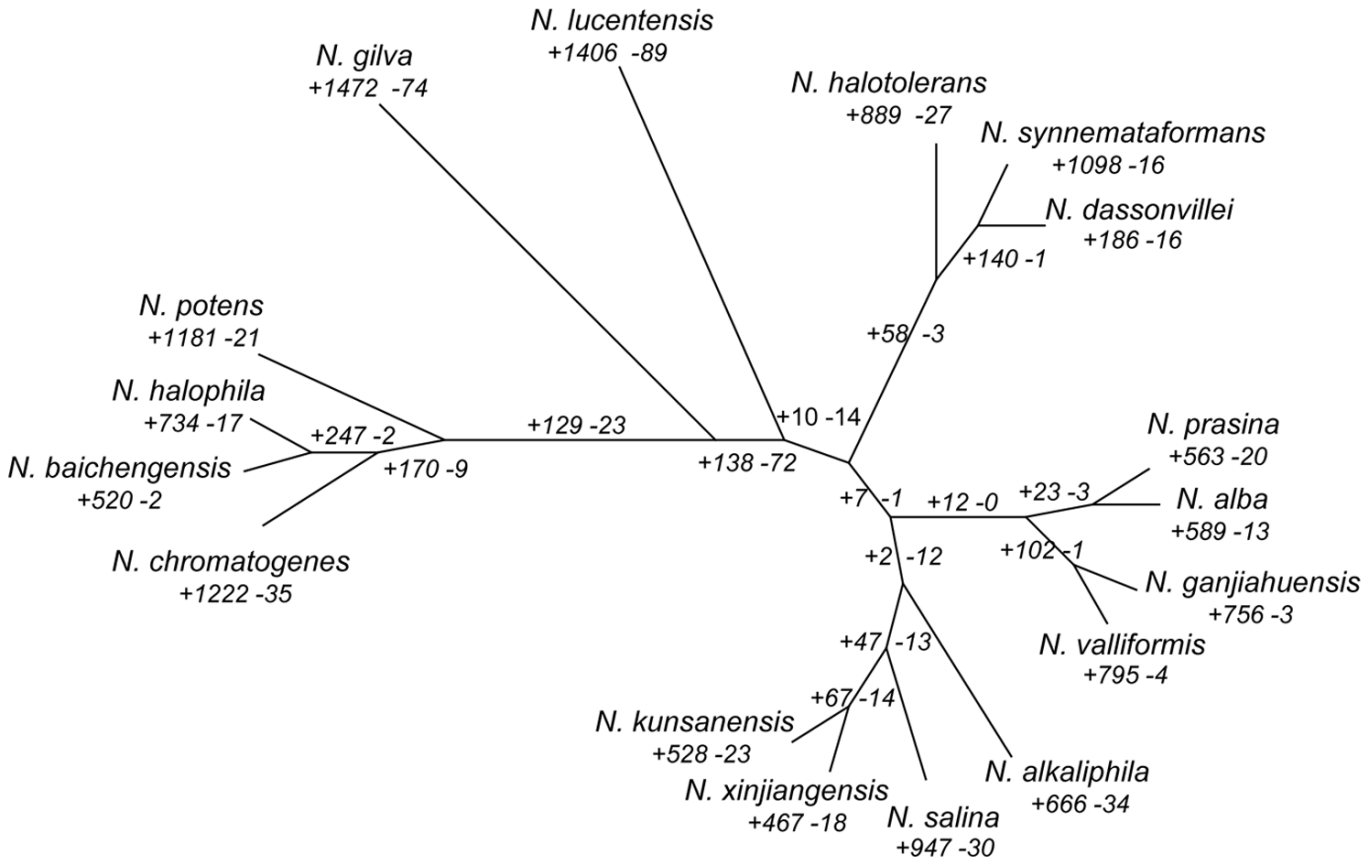
Gene content dendrogram and distribution of lineage-specific gene clusters. A dendrogram constructed by hierarchical clustering (UPGMA) based on dissimilarities in gene content (presence/absence of protein families) among the 17 species of *Nocardiopsis*. Dissimilarities were measured using Jaccard distance (one minus the Jaccard coefficient), ranging from 0 to 1. Numbers near each branch indicate corresponding events of homologous gene cluster acquisition (‘+’) and loss (‘–’). For example, 67 gene clusters were gained in the genome of the common ancestor of *N. kunsanensis* and *N. xinjiangensis* and do not contain a homolog in the other 15 genomes analyzed. Similarly, 14 gene clusters were missing in that ancestor’s genome but are present in all other 15 genomes.

We reconstructed a phylogenetic tree based on a super-matrix of amino acid sequences inferred from the 1,555 core orthologous clusters that were present as single-copy proteins in all 17 proteomes (Figure 3) and a dendrogram constructed by hierarchical clustering based on dissimilarities in gene content (Figure 4; Table S5). Both the super-matrix tree and the gene content dendrogram indicated that *N. synnemataformans* was the nearest neighbor of *N. dassonvillei* subsp. *dassonvillei. Nocardiopsis synnemataformans* was isolated from the sputum of a kidney transplant patient, and its pathogenicity has so far not been verified [2]. *Nocardiopsis dassonvillei* subsp. *dassonvillei* has frequently been isolated from patients and is implicated in cutaneous, pulmonary, eye, and diverse infections [3]. *Nocardiopsis valliformi*s and *N. ganjiahuensis* also formed a sister group. Both were isolated from alkaline lake sediments and cannot grow at pH 7.0 or lower. *Nocardiopsis valliformis* grows at a broad range of alkaline pHs, ranging from pH 8.0–14.0, with an optimum of pH 9.5–13.0 [43], while *N. ganjiahuensis* grows at pH 8.5–13.0, with an optimum of pH 8.5–9.5 [44]. *Nocardiopsis kunsanensis* and *N. xinjiangensis* were grouped together and shared an ancestor with *N. salina*. All three species are moderately halophilic actinomycetes isolated from saline sediment samples that can grow in the presence of 3–20% (w/v) NaCl; their optimum NaCl concentration is 10% [45].

The super-matrix tree and gene content dendrogram also showed some topological differences: *N. dassonvillei* subsp. *dassonvillei*, *N. synnemataformans*, *N. halotolerans,* and *N. lucentensis* formed a clade in the tree, while *N. lucentensis* was positioned by itself in the dendrogram. *Nocardiopsis prasina*, *N. ganjiahuensis*, *N. valliformis*, *N. alba*, and *N. alkaliphila* clustered together in the tree, while *N. alkaliphila* was more closely associated with *N. kunsanensis*, *N. xinjiangensis*, and *N. salina* in the dendrogram. These differences indicated that the *Nocardiopsis* gene repertoire reflected not only vertical inheritance of genes, but probable instances of one or more lineage-specific gene losses, non-orthologous gene displacements, or gene gains through horizontal transfer [46]. We expected horizontal gene transfer between phylogenetically-distant organisms and lineage-specific gene loss to have greater influences on the gene content-based phylogenetic analysis than the orthologous protein-based analysis [47]. Both the super-matrix tree and the gene content dendrogram highlighted that genetic exchange (e.g., gene acquisition and deletion) occurs often among the closely-related species, presumably because there are few ecological barriers between them.

**Figure 5:**
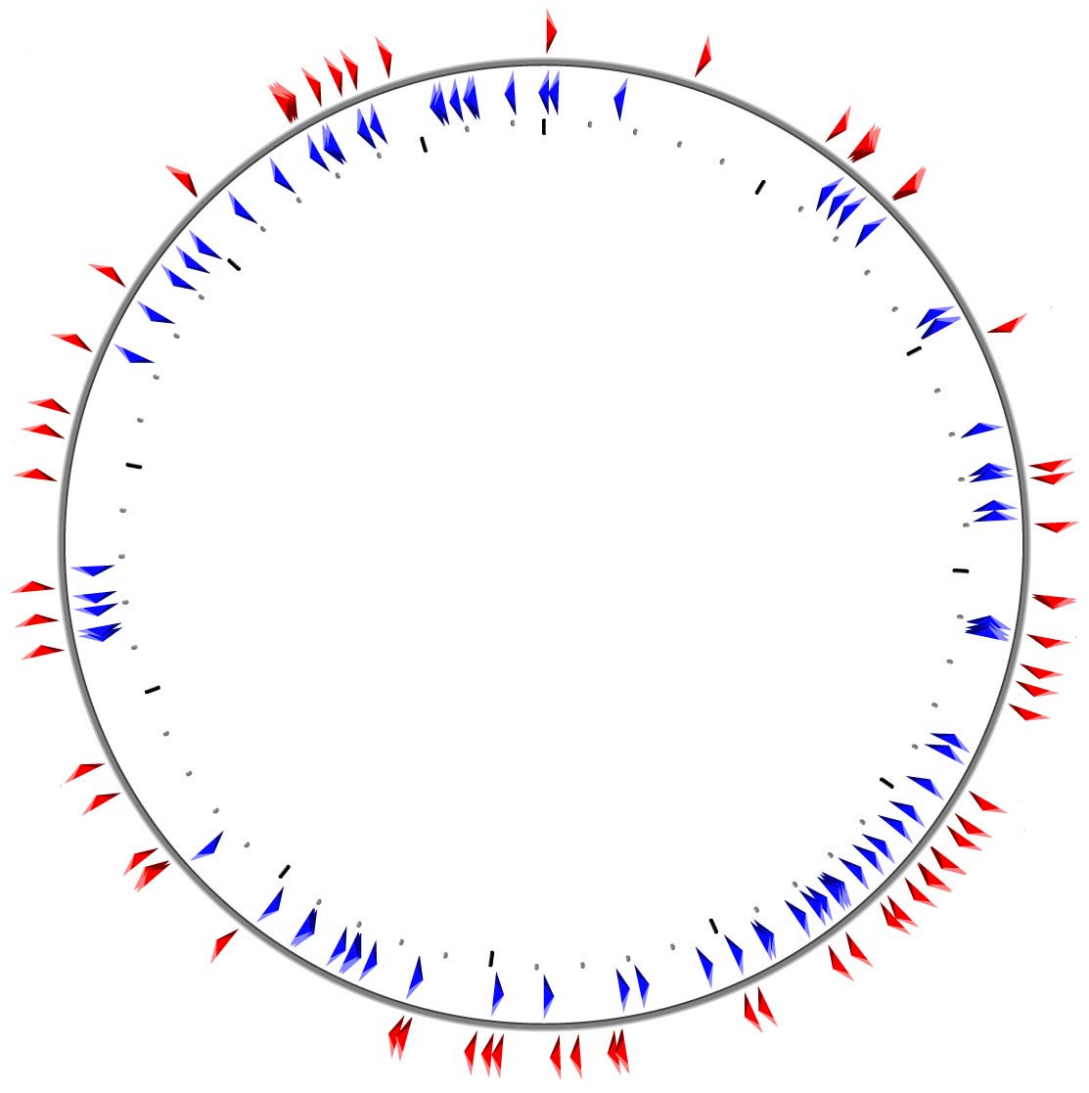
Distribution of species-specific genes on the chromosome of **Nocardiopsis dassonvillei** subsp. *dassonvillei*. Species-specific genes were dispersed on the chromosome. Thirty-five genes also randomly present on the plasmid pNDAS01 are not shown (see Figure S2). Blue/inside: genes on forward strand, Red/outside: genes on reverse strand.

Using the gene content dendrogram as a foundation, we classified the homologous clusters according to their presence/absence patterns in each of the selected genomes. Homologous clusters that could be explained by a single gene gain or loss event were counted and mapped on the phylogeny (Figure 4). Our phylogenetic approach showed that each genome had gained species-specific genes, ranging from a maximum of 1,472 in *N. gilva* to a minimum of 186 in *N. dassonvillei* subsp. *dassonvillei.* Similarly, each genome had lost genes specifically, from as many as 89 in *N. lucentensis* to just two in *N. baichengensis.* The genomic distribution of these species-specific genes was random throughout the chromosomes (Figure 5; Figure S2), indicating that these species-specific gene gains have been undergoing long-term evolution. In the common ancestor of *N. dassonvillei* subsp. *dassonvillei* and *N. synnemataformans*, both of which are frequently isolated from the human body, our phylogenetic approach identified 140 putative gene gains and one gene loss. Unfortunately, parts of these genes were poorly characterized, and their biological significance was difficult to infer based on available annotations. However, two gained genes that could be distinguished, a cystathionine beta-lyase (CBL) and a phenylacetic acid (PA) catabolic family protein, are reported to be essential for infection or survival in the host. CBL is a critical metabolite for bacterial pathogens [48], [49] that catalyzes the breakdown of cystathionine to homocysteine, the penultimate step in methionine biosynthesis. The CBL*-*encoding gene mutant of *Salmonella gallinarum* has an *in vivo* competitiveness defect when challenged, indicating that CBL is important for the virulence of *S. gallinarum* in chickens [48]. In addition, disruption of CBL-encoding gene was reported to attenuate virulence in *S. typhimurium* in a mouse model of systemic infection [49]. Furthermore, the components of biosynthetic pathways of sulfur-containing amino acids in general, and CBL in particular, are thought to be potential targets for the development of new antimicrobial agents [50]. The second identifiable gene belonged to a protein family protein involved in the catabolism of PA, which has been demonstrated to be required for bacterial pathogenicity. The *paaE* mutant of *Burkholderia cenocepacia* failed to survive in a rat model of infection [51]. Further studies have shown that the PA catabolic pathway is required for full pathogenicity of *B. cenocepacia* in the *Caenorhabditis elegans* host model [52]. Both genomes contained all gene clusters encoding PA catabolic genes, in which *paaABCDEFHIJK* was recognized as one operon and *paaZ* as another.

For the common ancestor of the two alkaliphilic taxa, *N. valliformis* and *N. ganjiahuensis*, we inferred 102 putative gene gains and one gene loss. Fifty-seven genes were inferred to be biologically significant based on available annotations. Thirteen of these proteins were transcriptional regulators, including members of the *LacI*, *LuxR*, *SARP*, *DeoR*, *XRE*, and *ArsR* families; five were transporters, including the *ABC* and *ABC-2* transporters and the extracellular solute-binding protein 5 family; five were proteases, including peptidase M16, peptidase M14, and periplasmic protease; and two were two-component system sensor kinases. Bacteria are frequently exposed to multiple stresses, such as pH changes, heat shock, and regular challenges by the host immune system. Thus, the maintenance of periplasmic proteins in a fully functional state is a challenge undertaken by the protein quality control system. Periplasmic proteases eliminate or refold damaged and unfolded proteins in the bacterial periplasm that are generated under conditions of stress [53].

A similar comparative genomic analysis identified 47 putative gene gains and 13 losses in the common ancestor of the three moderately halophilic taxa, *N. kunsanensis, N. xinjiangensis*, and *N. salina.* The set of 47 gained proteins included transcriptional regulators, glycosyltransferase, diaminopimelate decarboxylase, phytoene dehydrogenase, cyclic nucleotide-binding protein, adenylate and guanylate cyclase, peptidase U34, heat shock protein 20 (HSP20), phosphotransferase system EIIC, and parts of hypothetical proteins. Small heat-shock proteins are members of a diverse family of stress proteins that protect proteins under stressful conditions. Furthermore, HSP20 is important in coping with heat and osmotic stress in bifidobacterium, which has the highest level of activation of *hsp20* upon heat or osmotic shock so far reported among chaperone-encoding genes [54]. The set of 13 lost proteins included an alpha/beta hydrolase, protein-L-isoaspartate (D-aspartate) O-methyltransferase, aldehyde dehydrogenase, oligosaccharide biosynthesis Alg14-like protein, MOSC domain-containing protein, peptidase S9 prolyl oligopeptidase active site domain protein, band 7 protein, and other hypothetical proteins. Interestingly, band 7 protein is an integral membrane protein that is involved in membrane-associated processes, including ion channel function [55]. All these data indicated that osmotic regulation is a multigenic process resulting from numerous gene combinations and with multiple redundancies and that no genes were specifically shared by *N. kunsanensis, N. xinjiangensis*, and *N. salina* for osmotic regulation.

### Conclusions

*Nocardiopsis* is a group of widely-distributed actinobacteria that can populate quite varied ecological niches with differences in nutrients, osmotic pressure, pH, temperature, and the presence of toxic molecules. These factors can make their living conditions far from optimal. Our analysis of 17 *Nocardiopsis* species revealed that the key to such high versatility and adaptability may be their intrinsic genetic features. In the top ten core paralogous proteins, transcription regulators were the most common, and interestingly, the distribution of these families was quite uneven. The TerR and XRE families, accounting for about 40% of the transcription regulators, are implicated in antibiotic biosynthesis, efflux pumps, and osmotic stress, and in plasmid copying, bacteriophage transcription control, and methylation, respectively. ABC transporters and MFS genes were also very abundant, ensuring that *Nocardiopsis* can frequently exchange substances with the external environment. Several polyketide synthases, non-ribosomal peptide synthetases, and cytochrome P450 all improve organismal viability. Unlike intracellular bacteria, for which gene loss is a major force during reductive evolution [56], [57], *Nocardiopsis* species continuously acquire genes and expand their genomes to cope with environmental pressures. The core genome comprises at least 2,517 genes, approximately 43% of the genome content of each *Nocardiopsis* species studied. The open pan-genome consists of more than 22,000 genes, indicating that continued sequencing should identify about 755 novel genes per genome. Phenotype acquisition, species differentiation, and ecological flexibility have been created through a long evolutionary process in close contact with the niche. The inherent features of *Nocardiopsis* species – a dynamic genome with core and accessory proteins – have driven them to disperse widely and adapt to diverse local conditions.

## Materials and Methods

### Genome Sequencing

Sixteen genomes of the type strains of *Nocardiopsis*, including *N. alba* DSM 43377^T^ (GenBank accession number: ANAC00000000), *N. alkaliphila* YIM 80379^T^ (ANBD00000000), *N. baichengensis* YIM 90130^T^ (ANAS00000000), *N. chromatogenes* YIM 90109^T^ (ANBH00000000), *N. ganjiahuensis* DSM 45031^T^ (ANBA00000000), *N. gilva* YIM 90087^T^ (ANBG00000000), *N. halophila* DSM 44494^T^ (ANAD00000000), *N. halotolerans* DSM 44410^T^ (ANAX00000000), *N. kunsanensis* DSM 44524^T^ (ANAY00000000), *N. lucentensis* DSM 44048^T^ (ANBC00000000), *N. potens* DSM 45234^T^ (ANBB00000000), *N. prasina* DSM 43845^T^ (ANAE00000000), *N. salina* DSM 44839^T^ (ANBF00000000), *N. synnemataformans* DSM 44143^T^ (ANAW00000000), *N. valliformis* DSM 45023^T^ (ANAZ00000000), and *N. xinjiangensis* YIM 90010^T^ (ANBE00000000), were sequenced as part of this study, using a HiSeq 2000 sequencer (Illumina, San Diego, CA, USA) at BGI Shenzhen, China. The paired-end reads were assembled using SOAPdenovo [58]. Gene prediction was determined using Glimmer v. 3.0 [59]. The G+C contents (mole percent) were calculated from the genome sequences. The *N. dassonvillei* subsp. *dassonvillei* DSM 43111^T^ genome sequence was obtained from the National Center for Biotechnology Information site (ftp://ftp.ncbi.nlm.nih.gov/).

### Core and Pan-genome Analyses

Protein-coding sequences were retrieved from the genome sequences. Genes were identified using the program Inparanoid v. 2.0 [60], based on a BLAST score cut-off of 50 bits, an overlap cut-off of 50%, a BLOSUM45 amino acid substitution matrix, and a confidence value of 95% when searching for in-paralogs. MultiParanoid software was then used to cluster orthologs and in-paralogs shared by more than two species [61]. This approach yielded 22,143 homologous clusters, including 14,019 clusters containing a protein unique to one of the 17 genomes (Table S2). Functions were assigned to each protein family using the COG, JCVI, KEGG, SEED, Pfam, Swiss-Prot, TrEMBL, and Gene Ontology (GO) databases. The resulting binary gene content data (presence or absence of each protein family) is shown in Table S5.

Tables containing the complete data set were complied, and then core and pan genomes and unique genes were determined as previously described [25], [34]. Gene accumulation curves describing the number of new genes and genes in common, with the addition of new comparative genomes were performed using R (R Foundation for Statistical Computing, Vienna, Austria), based on the median value (Figure 2). This procedure was repeated 1,000 times by randomly modifying genome insertion order to obtain median and quartile values.

### Functional Classification of Homologous Clusters

Each core protein sequence in the 17 *Nocardiopsis* species was used as a query in BLAST [62] to search for homologous proteins in the Transporter Classification Database (TCDB) [63]. In addition, each protein sequence was scanned with HMMTOP [64] to predict the number of putative transmembrane segments (TMSs). On the basis of the numbers and locations of TMSs and sequence similarity, transport proteins were classified into homologous families and subfamilies according to the classification system in TCDB.

### Phylogenomic Analysis

Based on the homologous protein identifications, we selected a set of single-copy proteins shared by all 17 proteomes from which to infer the organismal phylogeny. Homologous protein clusters that contained more than one protein from any one proteome were excluded to avoid the complications introduced by paralogous clusters. Of the 22,143 homologous clusters, 1,555 were present in only a single copy in each of the 17 proteomes (i.e., had no paralogs). This set of 1,555 single-copy core proteins comprised putative orthologous genes. For each orthologous cluster, protein sequences were aligned using ClustalW [65]. The resulting alignments of individual proteins were concatenated and used to infer the organismal phylogeny using the Neighbor-Net algorithm in the package SplitsTree [66].

Hierarchical clustering (unweighted pair group method with arithmetic mean [UPGMA]) of the 17 genomes was performed using the distance between paired genomes based on gene content (presence/absence of each protein) measured by Jaccard distance (one minus the Jaccard coefficient).

Using the gene content dendrogram as a foundation, we categorized the homologous clusters according to their presence/absence patterns in the genomes. Homologous clusters that could be explained by a single gene gain or loss event were counted and mapped on the phylogeny. To check whether inferred gene losses were artifacts introduced by mis-annotation, we used all protein sequences in each homologous cluster as queries in BLASTP [63] searches against the complete genome sequence using a less stringent e-value cutoff of 1×10^–5^. For functional categorization, all protein sequences were used as queries in a first-pass automatic annotation utilizing the KAAS tool [67] provided by the KEGG database. The KEGG orthology assignments were further mapped onto the COG functional category assignments. Circular genome maps were generated using the Circular Genome Viewer [68].

## Supporting Information

Figure S1 **Phylogenetic tree of 17 species within the genus** ***Nocardiopsis.*** These organisms were distributed dispersively and well represented distribution of the genus. The tree was inferred from 1,306 aligned characters of the 16S rRNA gene sequence under the Neighbour-joining tree. Bootstrap values (expressed as percentages of 1000 replications) large than 50% were given at the nodes. Bar 1 nt substitution per 200 nt. Words in red represent species in this study.
(TIF)

Figure S2 **Distribution of species-specific genes on the plasmid pNDAS01 of** ***Nocardiopsis dassonvillei*** **subsp.** ***dassonvillei*** **.** The species-specific genes were found to randomly map on plasmid pNDAS01. Blue/inside: genes on forward strand. Red/outside: genes on reverse strand.
(TIF)

Table S1 **Genomic features and core and unique proteins for 17 species of** ***Nocardiopsis*** **.**
(XLS)

Table S2 **Complete list of the 22,143 homologous clusters in 17** ***Nocardiopsis*** **genomes.**
(XLS)

Table S3 **Distribution of homologous clusters among functional categories in** ***Nocardiopsis*** **genomes.**
(XLS)

Table S4 **Distribution of ABC transport family among core paralogous proteins in** ***Nocardiopsis*** **genomes.**
(XLS)

Table S5 **Complete list of the 22,143 homologous clusters (binary data for presence or absence of protein families) in** ***Nocardiopsis*** **genomes.**
(XLS)
