# Supplementary figures and images for: Comparative Genomic Analysis of the Genus Nocardiopsis Provides New Insights into Its Genetic Mechanisms of Environmental Adaptability
Source: PLoS One. 2013 Apr 23;8(4):e61528. doi: 10.1371/journal.pone.0061528 (PMC3634020; doi:10.1371/journal.pone.0061528)

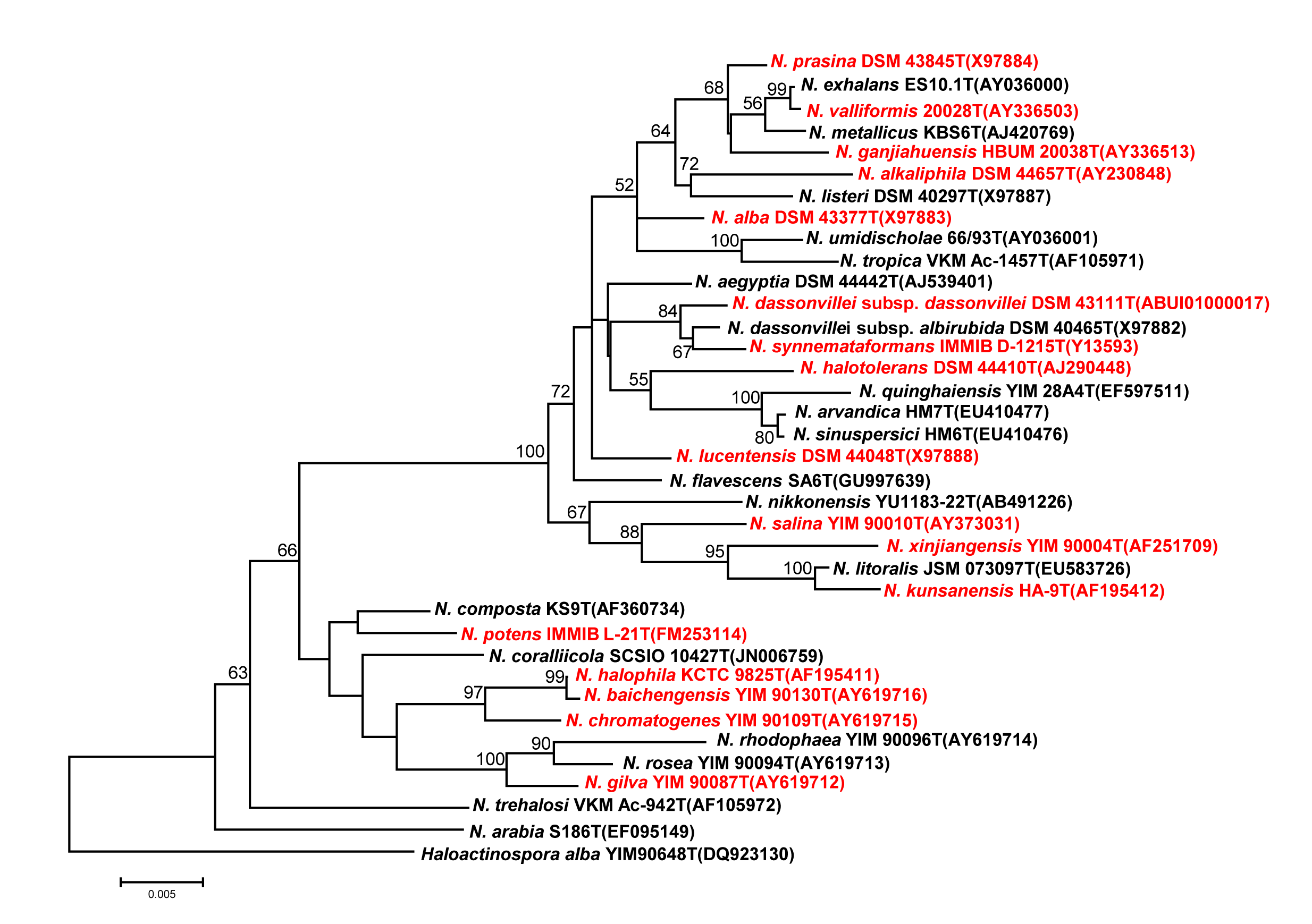

Supplement: Figure S1 — Phylogenetic tree of 17 species within the genus Nocardiopsis. These organisms were distributed dispersively and well represented distribution of the genus. The tree was inferred from 1,306 aligned characters of the 16S rRNA gene sequence under the Neighbour-joining tree. Bootstrap values (expressed as percentages of 1000 replications) large than 50% were given at the nodes. Bar 1 nt substitution per 200 nt. Words in red represent species in this study. (TIF) [file pone.0061528.s001.tif]

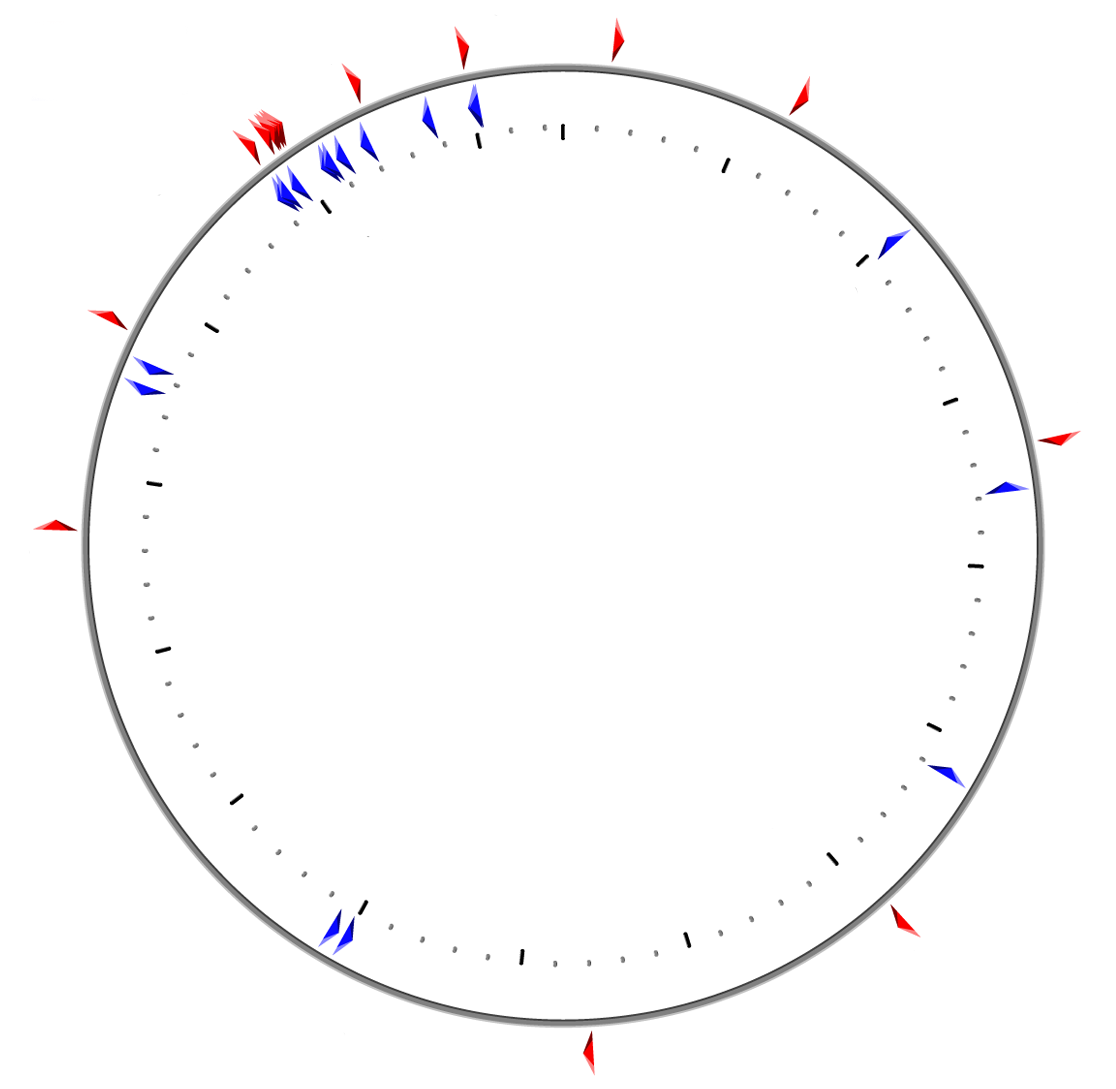

Supplement: Figure S2 — Distribution of species-specific genes on the plasmid pNDAS01 of Nocardiopsis dassonvillei subsp. dassonvillei . The species-specific genes were found to randomly map on plasmid pNDAS01. Blue/inside: genes on forward strand. Red/outside: genes on reverse strand. (TIF) [file pone.0061528.s002.tif]
